# Genetic and Infectious Profiles Influence Cerebrospinal Fluid IgG Abnormality in Japanese Multiple Sclerosis Patients

**DOI:** 10.1371/journal.pone.0095367

**Published:** 2014-04-15

**Authors:** Satoshi Yoshimura, Noriko Isobe, Takuya Matsushita, Katsuhisa Masaki, Shinya Sato, Yuji Kawano, Hirofumi Ochi, Jun-ichi Kira

**Affiliations:** 1 Department of Neurology, Neurological Institute, Graduate School of Medical Sciences, Kyushu University, Fukuoka, Japan; 2 Department of Molecular and Genetic Medicine, Ehime University Graduate School of Medicine, Ehime, Japan; Research Inst. of Environmental Med., Nagoya Univ., Japan

## Abstract

**Background:**

Abnormal intrathecal synthesis of IgG, reflected by cerebrospinal fluid (CSF) oligoclonal IgG bands (OBs) and increased IgG index, is much less frequently observed in Japanese multiple sclerosis (MS) cohorts compared with Western cohorts. We aimed to clarify whether genetic and common infectious backgrounds influence CSF IgG abnormality in Japanese MS patients.

**Methodology:**

We analyzed *HLA-DRB1* alleles, and IgG antibodies against *Chlamydia pneumoniae*, *Helicobacter pylori*, Epstein-Barr virus nuclear antigen (EBNA), and varicella zoster virus (VZV) in 94 patients with MS and 367 unrelated healthy controls (HCs). We defined CSF IgG abnormality as the presence of CSF OBs and/or increased IgG index (>0.658).

**Principal Findings:**

CSF IgG abnormality was found in 59 of 94 (62.8%) MS patients. CSF IgG abnormality-positive patients had a significantly higher frequency of brain MRI lesions meeting the Barkhof criteria compared with abnormality-negative patients. Compared with HCs, CSF IgG abnormality-positive MS patients showed a significantly higher frequency of DRB1*1501, whereas CSF IgG abnormality-negative patients had a significantly higher frequency of DRB1*0405. CSF IgG abnormality-positive MS patients had a significantly higher frequency of anti-*C. pneumoniae* IgG antibodies compared with CSF IgG abnormality-negative MS patients, although there was no difference in the frequency of anti-*C. pneumoniae* IgG antibodies between HCs and total MS patients. Compared with HCs, anti-*H. pylori* IgG antibodies were detected significantly less frequently in the total MS patients, especially in CSF IgG abnormality-negative MS patients. The frequencies of antibodies against EBNA and VZV did not differ significantly among the groups.

**Conclusions:**

CSF IgG abnormality is associated with Western MS-like brain MRI features. DRB1*1501 and *C. pneumoniae* infection confer CSF IgG abnormality, while DRB1*0405 and *H. pylori* infection are positively and negatively associated with CSF IgG abnormality-negative MS, respectively, suggesting that genetic and environmental factors differentially contribute to MS susceptibility according to the CSF IgG abnormality status.

## Introduction

Multiple sclerosis (MS) is a chronic inflammatory demyelinating disease of the central nervous system (CNS) with a supposed autoimmune origin involving T and B cells [1]. Interplay between genetic and environmental factors is assumed to contribute to the pathogenesis of MS [2]. The largest genetic effect on MS susceptibility is conferred by the major histocompatibility complex class II genes. In Caucasians, the HLA-DRB1*1501 allele is most strongly associated with MS, whereas the class I allele HLA-A*0201 allele appears to be a protective allele [3]. In the Japanese population, we and others reported that conventional MS (CMS) is associated with HLA-DRB1*1501, while opticospinal MS (OSMS) is associated with HLA-DPB1*0501 [4], [5], but no associations were found with any HLA class I alleles [6]. Recently, we reported that HLA-DRB1*0405 and HLA-DPB1*0301 are susceptibility alleles, while DRB1*0901 and DPB1*0401 are protective alleles for Japanese MS when neuromyelitis optica (NMO) and NMO spectrum disorder (NMOSD) patients are excluded [7].

Among many potential environmental risk factors, infection is likely to play a significant role in the acquisition of MS susceptibility or resistance. One candidate infectious agent is Epstein-Barr virus (EBV), which is more prevalent in Caucasian MS patients than in healthy controls (HCs), and therefore considered to increase susceptibility to MS [8], [9]. We recently found that the EBV infection rate has increased in a certain subgroup of Japanese MS patients not harboring HLA-DRB1*0405, a genetic risk factor for MS in the Japanese population, compared with HCs [7]. Since the first possible reported association between *Chlamydia pneumoniae* infection and MS [10], the significance of *C. pneumoniae* infection in MS has remained a matter of debate. We demonstrated that the anti-*C. pneumoniae* antibody positivity rate did not differ significantly between MS and HCs in Japanese [7], which is consistent with recent meta-analysis results [11]. We also found that the anti-*Helicobacter pylori* antibody positivity rate was lower among CMS patients than among HCs and OSMS patients in Japanese [12]. By contrast, the anti-*H. pylori* and anti-*C. pneumoniae* antibody positivity rates were increased in Japanese patients with NMO, especially in those with anti-aquaporin 4 (AQP4) antibodies [13], [14].

Abnormal intrathecal synthesis of IgG, reflected by cerebrospinal fluid (CSF) oligoclonal IgG bands (OBs) and increased IgG index, is a significant diagnostic hallmark in MS. More than 90% of MS patients are positive for CSF OBs in Western countries [15], [16]. However, this proportion appears to vary with ethnicity or geographical location, ranging from only 21–56% in Asian countries [17]–[21]. The presence of genetic influences on the OB phenotype is suggested by their associations in several populations. For example, the HLA-DRB1*15 allele is associated with OB-positive MS [19], [22], [23] and the HLA-DRB1*04 allele is associated with OB-negative MS [19], [22]. Although the prognostic significance of OBs is conflicting, the absence of OBs predicted a relatively benign clinical course and lower disease severity in some early studies [24], [25], but not all [18], [26]–[29]. Focusing on MRI findings, some studies have postulated a potentially lower lesion load in OB-negative patients [25], [29].

With this background, we aimed to investigate whether genetic and common infectious profiles influence CSF IgG abnormality in Japanese MS patients. In the present study, we focused on *HLA-DRB1* loci that are associated with MS in several populations, including Japanese. Among the infectious factors, we chose *C. pneumoniae*, *H. pylori*, EBV, and varicella zoster virus (VZV) infections, which could be potential environmental risk or protective factors for MS.

## Methods

### Participants

Ninety-four patients examined at the Department of Neurology, Kyushu University Hospital from 2006 to 2010 were enrolled. MS was defined using the 2005 revised McDonald criteria for MS [30]. NMO was defined as cases fulfilling the 2006 revised criteria for NMO [31]. We regarded patients as having an NMOSD when they fulfilled either two absolute criteria plus at least one supportive criterion, or one absolute criterion plus more than one supportive criterion from the 2006 NMO criteria, as previously described [7], [14]. None of the MS patients met the above-mentioned NMO/NMOSD criteria. Patients with primary progressive MS were excluded from the study. Informed consent was obtained from the 94 patients as well as 367 unrelated HCs. Among the MS patients, 84 patients had RRMS and 10 had SPMS. The MS patients were clinically classified into two subtypes, conventional MS (CMS) and OSMS, as described previously [4]. There were 70 patients with CMS and 24 patients with OSMS. We collected demographic data from the patients by retrospective review of their medical records. These data included sex, age at onset, disease duration, Kurtzke's Expanded Disability Status Scale (EDSS) scores [32], annualized relapse rate, Progression Index [33], CSF OBs, IgG index, and brain MRI lesions meeting the Barkhof criteria for MS [34]. CSF IgG was tested in the acute phase in all cases. OBs were determined by isoelectric focusing, as the most sensitive method for OB determination [15], [20]. OBs were considered positive when they were only detected in CSF and comprised at least two bands. The IgG index represents (CSF IgG/serum IgG)/(CSF albumin/serum albumin). The IgG index was considered to be increased if it was >0.658 [4]. Among the 92 MS patients assayed for CSF OBs, 42 were OB-positive and 50 were OB-negative. Among the 90 MS patients whose IgG index was assayed, 42 had elevation and 48 did not. In this study, we defined CSF IgG abnormality as the presence of CSF OBs and/or increased IgG index. This study was approved by the Kyushu University Hospital Ethics Committee. All individuals involved in this study signed a written informed consent.

### MRI analysis

All MRI studies were performed using 1.5 T units (Magnetom Vision and Symphony; Siemens Medical Systems, Erlangen, Germany) as previously described [35]. Brain MRI lesions were evaluated according to the Barkhof criteria for MS [34].

### *HLA-DRB1* genotyping

The genotypes of the *HLA-DRB1* alleles from the subjects were determined by hybridization between the products of polymerase chain reaction amplification of the *HLA-DRB1* genes and sequence-specific oligonucleotide probes, as described previously [7].

### Anti-AQP4 antibody assay

The presence of anti-AQP4 antibodies was assayed as described previously [36], using green fluorescent protein (GFP)-AQP4 (M1 isoform) fusion protein-transfected human embryonic kidney (HEK) cells. Serum samples diluted 1∶4 were assayed for anti-AQP4 antibodies at least twice using identical samples, with the examiners blinded to the origin of the specimens. Samples that gave a positive result twice were deemed positive. When the judgment was equivocal, we measured the anti-AQP4 antibody levels using GFP-AQP4 (M23 isoform)-transfected HEK cells.

### Detection of anti-*H. pylori*, anti-*C. pneumoniae*, anti-VZV, and anti-EBV nuclear antigen (EBNA) IgG antibodies

Serum anti-*C. pneumoniae*, anti-*H. pylori*, anti-EBNA, and anti-VZV IgG antibodies were measured using commercial ELISA kits (Vircell, Granada, Spain) in accordance with the manufacturer's instructions, as described previously [13]. Each antibody index was determined by dividing the optical density (OD) values for the target samples by the OD values for cut-off control samples and then multiplying by ten. As recommended by the manufacturer, an ELISA test index value was considered positive if higher than 11, equivocal if between 9 and 11, and negative if less than 9. According to the manufacturer's instructions, the ELISAs used in the present study have 100% sensitivity and 83% specificity for *H. pylori* infection and 100% sensitivity and 93% specificity for *C. pneumoniae* infection. Samples with equivocal results were retested for confirmation, and if the samples were equivocal twice, they were considered negative.

### Statistical analyses

The phenotype frequencies of the *HLA-DRB1* alleles were compared using the chi-square test, or Fisher's exact probability test when the criteria for the chi-square test were not fulfilled. Uncorrelated p-values (p^uncorr^) were corrected by multiplying them by the number of comparisons, as indicated in the table footnotes (Bonferroni–Dunn's correction), to calculate the corrected p-values (p^corr^). Fisher's exact probability test was used to compare sex, brain MRI lesions meeting the Barkhof criteria [34], and frequencies of antibodies against common infectious agents among the groups. Other demographic features were analyzed using the Wilcoxon rank sum test. We analyzed the trends in the proportions of patients among subgroups with advancing year of birth using the Cochran–Armitage trend test. All analyses were performed using JMP 8.0.3 (SAS Institute, Cary, NC). In all assays, values of p<0.05 were considered statistically significant.

## Results

### Relationships between CSF IgG abnormality status and demographic features in MS

**Figure 1:**
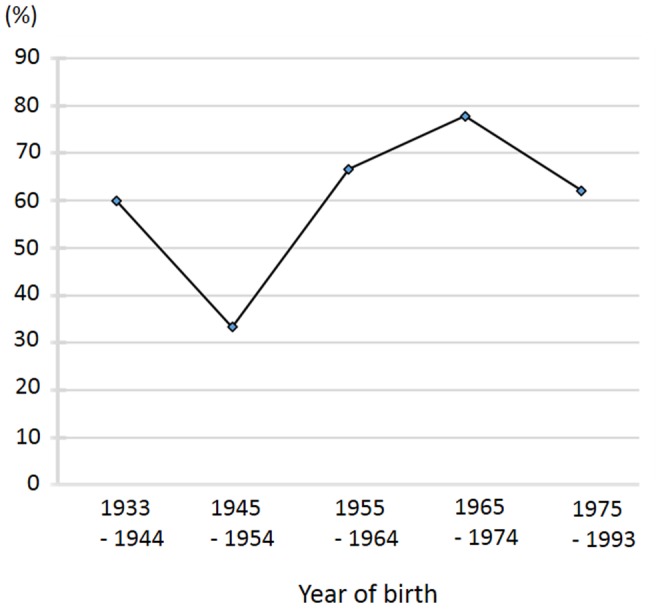
Proportions of patients with CSF IgG abnormality by year of birth. Among the MS patients, the proportion of patients with CSF IgG abnormality did not change significantly with advancing year of birth. CSF, cerebrospinal fluid; MS, multiple sclerosis.

Among the 94 MS patients, 59 (62.8%) were CSF IgG abnormality-positive and 35 were abnormality-negative. CSF IgG abnormality-positive patients had a significantly higher frequency of brain MRI lesions meeting the Barkhof criteria than abnormality-negative patients (46/58, 79.3% versus 16/34, 47.1%, p = 0.0025) (Table 1). The sex distribution, age at onset, disease duration, EDSS score, annualized relapse rate, Progression Index, and frequency of OSMS presentation with short spinal cord lesions (less than three vertebral segments) showed no associations with the presence or absence of CSF IgG abnormality (Table 1). The proportion of patients with CSF IgG abnormality did not change significantly with advancing year of birth (p>0.1) (Figure 1).

**Table 1:** Comparisons of the demographic features of MS patients according to the CSF IgG abnormality status.

	CSF IgG abnormality (+) (n = 59)	CSF IgG abnormality (−) (n = 35)	P value
Male:female	18∶41	15∶20	0.2669
Age at onset (years)^a^	30.54±12.12	34.06±15.70	0.4157
Disease duration (years)^a^	10.48±8.24	10.29±8.45	0.7899
EDSS score^a^	2.74±1.92	2.83±1.84	0.6420
Annualized relapse rate^a^	0.58±0.60	0.87±0.90	0.0946
Progression index^a^	0.39±0.39	0.90±2.00	0.4740
Barkhof criteria^b^	46/58 (79.3%)	16/34 (47.1%)	0.0025
OSMS with short spinal cord lesions^c^	13/59 (22.0%)	11/35 (31.4%)	0.3366

a Values represent the mean ± SD.

b Brain MRI lesions meeting the Barkhof criteria [34].

c Opticospinal form of MS with short spinal cord lesions extending less than three vertebral segments.

CSF, cerebrospinal fluid; EDSS, Kurtzke's Expanded Disability Status Scale; MS, multiple sclerosis.

### Correlation of CSF IgG abnormality according to clinical subtypes

The frequency of CSF IgG abnormality did not differ significantly between RRMS (52/84, 61.9%) and SPMS (7/10, 70%). Additionally, there was no significant difference in the frequency of CSF IgG abnormality between CMS (46/70, 65.7%) and OSMS (13/24, 54.2%).

### *HLA-DRB1* alleles in all MS patients

Compared with HCs, MS patients showed a significantly higher frequency of the DRB1*0405 allele (p^corr^ = 0.0196, OR  = 2.217, 95% CI  = 1.389–3.539) and a significantly lower frequency of the DRB1*0901 allele (p^corr^ = 0.0084, OR  = 0.279, 95% CI  = 0.135–0.575) (Table 2).

**Table 2:** Comparisons of the phenotype frequencies of the *HLA-DRB1* alleles.

DRB1*X	MS (n = 94)	CSF IgG abnormality (+) (n = 59)	CSF IgG abnormality (−) (n = 35)	HCs (n = 367)
0101 (%)	13 (13.8)	7 (11.9)	6 (17.1)	51 (13.9)
0301 (%)	0 (0.0)	0 (0.0)	0 (0.0)	2 (0.5)
0401 (%)	3 (3.2)	1 (1.7)	2 (5.7)	4 (1.1)
0403 (%)	4 (4.3)	4 (6.8)	0 (0.0)	18 (4.9)
0404 (%)	2 (2.1)	2 (3.4)	0 (0.0)	0 (0.0)
0405 (%)	42 (44.7)* ^a^	22 (37.3)	20 (57.1)* ^b^	98 (26.7)
0406 (%)	13 (13.8)	8 (13.6)	5 (14.3)	23 (6.3)
0407 (%)	0 (0.0)	0 (0.0)	0 (0.0)	2 (0.5)
0410 (%)	2 (2.1)	2 (3.4)	0 (0.0)	4 (1.1)
0701 (%)	0 (0.0)	0 (0.0)	0 (0.0)	2 (0.5)
0802 (%)	12 (12.8)	8 (13.6)	4 (11.4)	26 (7.1)
0803 (%)	11 (11.7)	9 (15.3)	2 (5.7)	58 (15.8)
0901 (%)	9 (9.5)* ^c^	6 (10.2)	3 (8.6)	101 (27.5)
1001 (%)	0 (0.0)	0 (0.0)	0 (0.0)	4 (1.1)
1101 (%)	4 (4.3)	2 (3.4)	2 (5.7)	16 (4.4)
1106 (%)	0 (0.0)	0 (0.0)	0 (0.0)	1 (0.3)
1201 (%)	9 (9.6)	6 (10.2)	3 (8.6)	33 (9.0)
1202 (%)	0 (0.0)	0 (0.0)	0 (0.0)	13 (3.5)
1301 (%)	1 (1.1)	1 (1.7)	0 (0.0)	1 (0.3)
1302 (%)	3 (3.2)	1 (1.7)	2 (5.7)	49 (13.4)
1403 (%)	2 (2.1)	0 (0.0)	2 (5.7)	8 (2.2)
1405 (%)	3 (3.2)	2 (3.4)	1 (2.9)	14 (3.8)
1406 (%)	3 (3.2)	2 (3.4)	1 (2.9)	8 (2.2)
1454 (%)	2 (2.1)	2 (3.4)	0 (0.0)	19 (5.2)
1501 (%)	24 (25.5)	20 (33.9)* ^d^	4 (11.4)	60 (16.4)
1502 (%)	14 (14.9)	8 (13.6)	6 (17.1)	80 (21.8)
1601 (%)	0 (0.0)	0 (0.0)	0 (0.0)	1 (0.3)
1602 (%)	0 (0.0)	0 (0.0)	0 (0.0)	3 (0.8)

*^a^Compared with HCs, p^corr^ = 0.0196, OR  = 2.217, 95% CI  = 1.389–3.539.

*^b^Compared with HCs, p^corr^ = 0.0056, OR  = 3.660, 95% CI  = 1.802–7.431.

*^c^Compared with HCs, p^corr^ = 0.0084, OR  = 0.279, 95% CI  = 0.135–0.575.

*^d^Compared with HCs, p^corr^ = 0.0392, OR  = 2.624, 95% CI 1.432–4.809.

p^uncorr^ was corrected by multiplying the value by 28 to calculate p^corr^.

CI, confidence interval; CSF, cerebrospinal fluid; HCs, healthy controls; MS, multiple sclerosis; OR, odds ratio; p^corr^, corrected p value.

### *HLA-DRB1* alleles in MS patients according to the presence or absence of CSF IgG abnormality

Compared with HCs, CSF IgG abnormality-positive MS patients showed a significantly higher frequency of the DRB1*1501 allele (p^corr^ = 0.0392, OR  = 2.624, 95% CI  = 1.432–4.809), whereas CSF IgG abnormality-negative MS patients showed a significantly higher frequency of the DRB1*0405 allele (p^corr^ = 0.0056, OR  = 3.660, 95% CI  = 1.802–7.431) (Table 2). The frequency of CSF IgG abnormality was 83.3% in MS patients with the DRB1*1501 allele compared with 52.4% in MS patients with the DRB1*0405 allele (p = 0.0119).

### Relationships between CSF IgG abnormality status and common infectious agents

**Figure 2:**
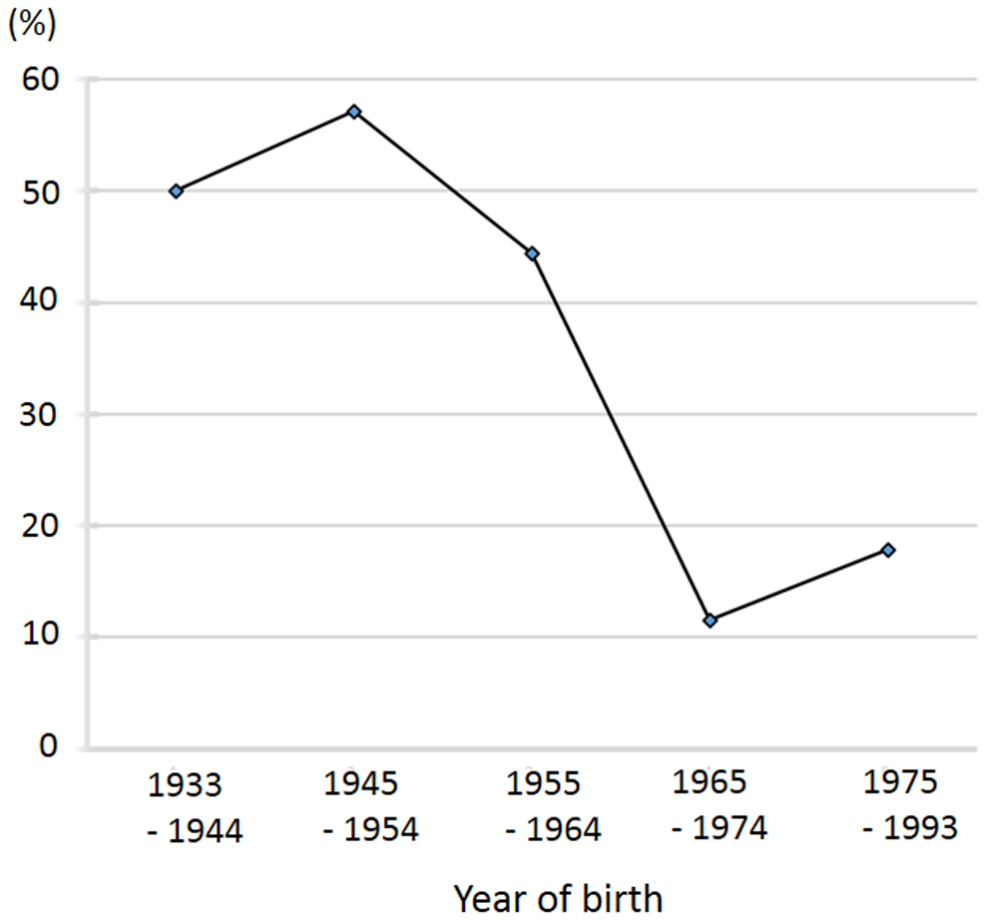
Proportions of patients with *Helicobacter pylori* infection by year of birth. Among the MS patients, the proportion of patients with *H. pylori* decreased markedly in those born after 1965. MS, multiple sclerosis.

Anti-*C. pneumoniae* IgG antibodies were significantly more frequently detected in MS patients with CSF IgG abnormality than in those without CSF IgG abnormality (p = 0.0119) (Table 3), although there was no difference in the frequency of anti-*C. pneumoniae* IgG antibodies between HCs and total MS patients. Compared with HCs, anti-*H. pylori* IgG antibodies were detected significantly less frequently in the total MS patients (p = 0.0451) and CSF IgG abnormality-negative MS patients (p = 0.0474) (Table 3). There was no difference in the frequency of anti-*H. pylori* IgG antibodies between CSF IgG abnormality-positive and abnormality-negative patients. Neither the anti-EBNA nor anti-VZV antibody positivity rates differed significantly among the groups. Among the common infectious agents, only the *H. pylori* infection rate decreased successively with advancing year of birth (p = 0.0014) (Figure 2).

**Table 3:** Comparisons of the frequencies of antibodies against common infectious agents.

	MS	CSF IgG abnormality (+)	CSF IgG abnormality (−)	HCs
*Chlamydia pneumoniae*	56/90 (62.22%)	42/58 (72.4%)*	14/32 (43.8%)*	92/156 (58.97%)
*Helicobacter pylori*	26/90 (28.89%)**	19/58 (32.8%)	7/32(21.9%)***	74/177 (41.81%)** ^,^ ***
EBV	86/90 (95.56%)	56/58 (96.6%)	30/32 (93.8%)	143/156 (91.67%)
VZV	89/90 (98.89%)	57/58 (98.3%)	32/32 (100%)	153/156 (98.08%)

*p = 0.0119, compared with IgG abnormality (−) MS patients.

**p = 0.0451, compared with HCs.

***p = 0.0474, compared with HCs.

*^,^**^,^***Significant difference between the linked values (p<0.05).

The age of the patients during examination did not differ significantly among HCs and MS patients, regardless of the presence or absence of IgG abnormality (mean ± SD in years: 37.21±12.54 for MS; 36.19±11.36 for IgG abnormality-positive MS; 39.00±14.39 for IgG abnormality-negative MS; and 38.93±12.11 for HCs).

CSF, cerebrospinal fluid; EBV, Epstein-Barr virus; HCs, healthy controls; MS, multiple sclerosis; p^corr^, corrected p value; VZV, varicella zoster virus.

## Discussion

The main new findings of the present study are as follows: (1) compared with HCs, CSF IgG abnormality-positive MS patients had a significantly higher frequency of HLA-DRB1*1501, whereas CSF IgG abnormality-negative MS patients had a significantly higher frequency of HLA-DRB1*0405; (2) CSF IgG abnormality-positive MS patients had a significantly higher frequency of anti-*C. pneumoniae* IgG antibodies compared with abnormality-negative MS patients, although there was no difference in the frequency of anti-*C. pneumoniae* IgG antibodies between HCs and total MS patients; and (3) compared with HCs, the frequencies of anti-*H. pylori* IgG antibodies were lower, especially in CSF IgG abnormality-negative MS patients.

The number of enrolled MS patients was not large because of the relative rarity of the disease in the Japanese population, and this could lead to partly inconclusive results. However, this study is the first to investigate the influence of *HLA-DRB1* alleles on CSF IgG abnormality in MS patients in Japan when NMO and NMOSD patients were excluded, and is the only study to simultaneously investigate the influence of common infectious agents as environmental risk or protective factors. The ELISAs used in the present study have reasonably high sensitivity and specificity [37], [38], although *H. pylori* and *C. pneumoniae* infections should be confirmed by methods other than ELISA in future studies.

In the present study, we demonstrated that the presence or absence of CSF IgG abnormality did not predict the prognosis for the disease course of Japanese MS patients, consistent with relatively larger studies in Western countries and a Japanese study in Hokkaido, the northernmost island of Japan [18], [26]–[28]. Additionally, the frequency of CSF IgG abnormality did not differ according to the clinical subtypes of MS.

In CSF IgG abnormality-positive MS patients, the only significant difference was the more frequent presence of brain MRI lesions meeting the Barkhof criteria compared with CSF IgG abnormality-negative MS patients. Carriage of HLA-DRB1*1501 was associated with an approximately 2.6-fold increased risk for CSF IgG abnormality-positive MS. This is in line with previous findings demonstrating that DRB1*15 is associated with OB-positive MS in Swedish patients [22], Spanish patients [23], and the Japanese population of Hokkaido [19]. Taken together, in this subpopulation, MS was associated with greater brain MRI lesion loads, presence of the HLA-DRB1*1501 allele, and increased humoral immune responses in CSF in Japanese. These features also resemble those of MS in Western people [39], [40]. Therefore, this subgroup of Japanese patients represents a “Western” type of MS in terms of CSF, neuroimaging, and genetic characteristics. In Caucasians, the presence of the DRB1*1501 allele promotes the development of more T2 lesions [41] and intrathecal IgG synthesis [42]. Similar biological mechanisms may occur in Asian patients.

In CSF IgG abnormality-negative MS patients, HLA-DRB1*0405 showed an approximately 3.6-fold increased risk for the condition. In this subgroup, MS was characterized by lower MRI brain lesion loads. This is in line with previous findings that DRB1*04 is associated with OB-negative MS in Swedish patients [22] and the Japanese population of Hokkaido [19]. The low frequency of CSF IgG abnormality is a unique feature in Japanese MS patients, compared with Western MS patients [17], [20]. DRB1*0405 is present in a relatively minor population of Caucasian MS patients [22], while about 60% of MS patients in Northern Europe are positive for HLA-DRB1*15, compared with 30% of HCs [43]. The relatively high frequency of Japanese MS patients carrying the DRB1*0405 allele may be partly responsible for the low prevalence of CSF IgG abnormality in Japanese MS patients. According to the fourth nationwide survey of MS in Japanese people, the most common type of MS had neither Barkhof brain lesions nor longitudinally extensive spinal cord lesions [44]. Hence, CSF IgG abnormality-negative Japanese MS could be a unique subgroup of MS in terms of CSF, neuroimaging, and genetic characteristics. Kuenz et al. [45] showed that CSF B cells were correlated with paraclinical markers such as high numbers of MRI T2 lesions, intrathecal IgG synthesis, and intrathecal production of MMP-9 and B cell chemokine CxCL-13. These findings may suggest that distinct immune mechanisms between IgG abnormality-positive and abnormality-negative patients lead to the differences in their CSF and neuroimaging characteristics.

In the present study, we showed that *C. pneumoniae* infection was higher in CSF IgG abnormality-positive MS patients than in abnormality-negative MS patients. The mechanism for the abnormal intrathecal IgG synthesis in MS patients infected with *C. pneumoniae* could be molecular mimicry [46], i.e., cross-reactivity of humoral immune responses against *C-pneumoniae* antigens and CNS self-antigens. To date, however, OBs have not been found to react highly specifically with any microbial antigens or self-antigens. It is possible that anti-*C. pneumoniae* IgG antibodies directly confer CSF IgG abnormality in MS patients. In general, however, CSF anti-*C. pneumoniae* IgG antibodies are only found in a small portion of MS patients, with no differences between MS and controls [47]–[50]. These findings suggest that *C*. *pneumoniae* infection may indirectly modify the intrathecal humoral immune functions, leading to CSF IgG abnormality in patients with MS.

It is interesting to note that CSF IgG abnormality-negative MS patients had a lower frequency of *H. pylori* infection compared with HCs in addition to a lower frequency of *C. pneumoniae* infection compared with CSF IgG abnormality-positive MS patients. To date, there have been no reports focusing on the association between CSF IgG abnormality and *H. pylori* infection, and there has been no firm evidence of molecular mimicry between human myelin antigens and *H. pylori*. Therefore, it is extremely difficult to speculate on the mechanism by which *H. pylori* infection differentially affects MS subpopulations with and without CSF IgG abnormality. However, these observations extend our previous finding that the rates of *H. pylori* infection, which occurs in the infantile period and reflects sanitary conditions in younger ages, were lower in Japanese MS patients, compared with HCs [12]. Additionally, the proportion of patients with *H. pylori* infection decreased successively with advancing year of birth. These findings collectively suggest that CSF IgG abnormality-negative MS patients may have grown up in a relatively clean environment. It is possible that a clean environment at younger ages may confer CSF IgG abnormality-negative MS. We previously reported that HLA-DRB1*0405-positive MS showing a lower frequency of CSF IgG abnormality is increasing in the younger Japanese population [7]. Thus, a modernized clean environment may potentiate susceptibility to this subtype of MS without CSF IgG abnormality in HLA-DRB1*0405 carriers. This possibility should be investigated in future large-scale studies.

In conclusion, DRB1*1501 and *C. pneumoniae* infection confer CSF IgG abnormality, while DRB1*0405 and *H. pylori* infection are positively and negatively associated with CSF IgG abnormality-negative MS, respectively, suggesting that genetic and environmental factors differentially contribute to MS susceptibility according to the CSF IgG abnormality status.
